# Health-related quality of life in first-ever stroke patients

**DOI:** 10.4103/0256-4947.51814

**Published:** 2009

**Authors:** Eda Gurcay, Ajda Bal, Aytul Cakci

**Affiliations:** From the Ministry of Health, Ankara, and Diskapi Yildirim Beyazit Education and Research Hospital, Department of Physical Therapy and Rehabilitation, Ankara, Turkey

## Abstract

**BACKGROUND AND OBJECTIVES:**

Health-related quality of life (HRQOL) is important to measure as it is an indication of outcome after stroke. Our objectives were to assess HRQOL in patients 3 months after stroke and to identify factors that predict HRQOL in stroke survivors.

**PATIENTS AND METHODS:**

This cross-sectional study included 67 first-ever stroke patients hospitalized in the Ministry of Health Ankara Diskapi Yildirim Beyazit Education and Research Hospital Physical Therapy and Rehabilitation Clinic. HRQOL was measured by means of the Stroke Impact Scale-16 (SIS-16). Patients were characterized by age, sex, duration of education, comorbidities, stroke type, affected side, concordance (paretic arm=dominant hand), cognitive function (Mini-Mental State Examination [MMSE]), and functional status (Functional Independence Measure [FIM]). We used a linear regression model to examine the influence of demographic and clinical characteristics on the different SIS-16 domains.

**RESULTS:**

The mean (SD) for age of the 67 patients was 62.03 (13.22) years (range, 33 to 81 years). The MMSE and FIM scores were significantly correlated with the SIS-16 score (*P*<.001). Linear regression analysis showed that age and functional status were the major independent determinants affecting HRQOL (*P*=.002 and *P*<.001, respectively).

**CONCLUSION:**

In this study, we found that age and functional status had a powerful influence on HRQOL. Comprehensive therapy programs aimed to improve HRQOL should focus on improving functional disability, particularly in older stroke patients. There is a need for long-term follow-up studies in stroke patients throughout all recovery stages to evaluate HRQOL in more detail.

Stroke is the leading cause of serious, long-term neurologic impairment and functional disability. Depending on the severity and type, a stroke can leave an individual with residual impairment of physical, psychological, social and cognitive functions.^1^–^5^ Health-related quality of life (HRQOL) covering physical, cognitive and social functions has been emphasized as an important index of outcome after stroke; therefore, its measurement is important. Several factors, including age, gender, dependency in activities of daily living (ADL)/disability, and decreased social support have been associated with poorer HRQOL in stroke survivors.^5^–^9^ The Short Form-36 (SF-36) has been widely used in many clinical trials in recent years to evaluate HRQOL after stroke; however, ceiling and floor effects limit the ability of these instruments to evaluate the stroke patient's disability prognoses or health outcomes over time.^10^,^11^ The SF-36 has shown floor effects whereas the ADL measures have shown ceiling effects, i.e., a large portion of patients are located in the highest or lowest possible score of the instrument, reducing the instrument's ability to detect change. To address these limitations, the Stroke Impact Scale (SIS), a stroke-specific outcome measure, a more comprehensive measure of health outcomes, was designed. The SIS version 3.0 includes 59 items and assesses 8 domains (strength, hand function, ADL/instrumental ADL, mobility, communication, emotion, memory, thinking and social participation). Sixteen items from 4 of the 8 domains can be combined into an overall physical component score. This composite domain of physical function includes 16 items and is referred to as the SIS-16.^12^–^14^

The main objectives of the present study were to assess the HRQOL in patients 3 months after stroke and to identify factors that predict HRQOL. Using the SIS-16, the study is a comprehensive analysis of the possible association between demographic characteristics (e.g., age, gender, education duration), clinical characteristics (e.g., stroke type, affected side), cognitive function, functional status and HRQOL.

## PATIENTS AND METHODS

This was a cross-sectional study of all eligible patients admitted to the Ministry of Health Ankara Diskapi Yildirim Beyazit Education and Research Hospital Physical Therapy and Rehabilitation Clinic between January 2007 and March 2008. Sixty-seven patients surviving 3 months after stroke participated in the study. Criteria for inclusion in the study were first-ever stroke (cerebral infarction or hemorrhage), confirmed by either brain CT or MRI findings consistent with the clinical presentation, patient willingness to participate, and the availability of a complete Mini-Mental State Examination (MMSE), Functional Independence Measure (FIM), and SIS-16 data. Exclusion criteria were stroke due to other intracranial diseases such as subarachnoid hemorrhage, sinus venous thrombosis and severe head trauma, absence of neuroimaging data, co-morbidities that would limit life expectancy, and severe cognitive impairment. All patients were informed about the nature of the study and provided informed consent prior to beginning the trial, which was conducted in accordance with the Helsinki Declarations of 1975.

Demographic data recorded included age, gender, duration of education, and comorbidities. Comorbidities (e.g., hypertension, diabetes mellitus, cardiovascular diseases) were defined as a known history reported by the stroke survivors and measured by summing the major health problems as no comorbidity, one comorbidity or two or more comorbidities. The stroke type (infarct/hemorrhage), affected side (right/left) and concordance (paretic arm=dominant hand) (discordant/concordant) were categorized in defining characteristics of the stroke. Cognitive function was assessed using the MMSE.15 The MMSE is a widely used, reliable and validated instrument used in screening for cognitive impairment, with scores ranging from 0 to 30 and a score <24 indicating cognitive impairment. The exam assesses aspects of cognition and is easily performed. Contents include orientation, attention, learning, calculation, abstraction, information, construction and delayed recall. Functional status was measured by scores on the motor and cognitive components of the FIM.^16^,^17^ It is an 18-item instrument graded on a 7-point ordinal scale, with a maximum total score of 126. HRQOL was assessed using the SIS-16. The SIS-16, as previously described, includes four domains (strength, hand function, ADL/instrumental ADL, and mobility) to assess physical function.^11^,^14^,^18^

The respondent answered with either the number or the text associated with the number (e.g., “5” or “Not difficult at all”; “1” or “could not do at all”) for an individual question. These four domains were aggregated to create one physical domain and were transformed to a scale with total scores ranging from 0 to 100, with 0 being the worst possible score and 100 the best possible score. As such, the floor effect is defined as a score of 0, indicating that patients are unable to perform physical functioning, and the ceiling effect is a score of 100, indicating that patients are able to perform all physical activities.

Data were analyzed with SPSS 15.0 (SPSS Inc., Chicago, USA) software. Descriptive data were shown as the mean and standard deviation or as frequency tables. The independent samples t test or Mann-Whitney U test (following the Kolmogorov-Smirnov test of normality) were used to compare the SIS-16 values of the two groups. Differences among the groups in more than two categories were investigated by the one-way ANOVA test. A Pearson correlation was calculated for the relationships among the continuous variables (age, education duration, MMSE, FIM). Effects of independent variables (for continuous variables) on the dependent variable (SIS-16) were estimated by multiple step-wise linear regression analysis. *P* values less than or equal to .05 were considered statistically significant.

## RESULTS

Of the 70 patients eligible for the study, 3 patients did not agree to participate. The age of the remaining 67 survivors ranged from between 33 to 81 years (Table 1). On average, participants had a mean (SD) of 1.25 (0.82) comorbidities. Forty-one patients had non-dominant hand involvement. Other demographic and clinical characteristics of the patients are presented in Table 1. The SIS-16 score showed no significant differences related to sex, comorbidity, stroke type, affected side or concordance (Table 2). The MMSE and FIM scores were significantly correlated with the SIS-16 score (*P*<.001). In the linear regression analysis, the SIS-16 score was the dependent variable. Age and FIM score were independently associated with the SIS-16 (*P*=.002 and *P*<.001, respectively) (Table 3). For each one unit increase in age, the SIS-16 score showed a 0.226 unit decrease and for each one unit increase in the FIM score, the SIS-16 score showed a 0.658 unit increase. The SIS-16 score was not influenced by duration of education or the MMSE score (*P*>.05).

**Table 1:** Demographic and clinical characteristics of stroke survivors (n=67).

Characteristic	
Age (years), mean (SD)	62.03 (13.22)
Gender, n (%)	
Male	36 (53.7)
Female	31 (46.3)
Education duration (years), mean (SD)	4.58 (3.92)
Comorbidity, n (%)	
None	11 (16.4)
One	28 (41.8)
Two or more	28 (41.8)
Stroke type, n (%)	
Infarct	55 (82.1)
Hemorrhage	12 (17.9)
Affected side, n (%)	
Right	27 (40.3)
Left	40 (59.7)
Concordance, n (%)	
Discordant	41 (61.2)
Concordant	26 (38.8)
MMSE, mean (SD)	22.07 (6.53)
FIM, mean (SD)	91.37 (28.22)
SIS-16, mean (SD)	45.74 (24.71)

MMSE: Mini-Mental State examination, FIM: Functional Independence Measure, SIS-16: Stroke Impact Scale-16

**Table 2:** Comparisons of the SIS-16 values according to patient characteristics.

	Mean (SD)	*P*
Sex		
Male	44.58 (25.87)	.683
Female	47.08 (23.65)	
Comorbidity		
None	54.55 (21.45)	.194
One	39.79 (26.24)	
Two or more	48.21 (23.65)
Stroke type		
Infarct	45.43 (24.98)	.800
Hemorrhage	47.14 (24.47)	
Affected side		
Right	43.0 (23.24)	.461
Left	47.58 (25.78)	
Concordance		
Discordant	47.56 (25.51)	.451
Concordant	42.85 (23.60)	

**Table 3:** Multiple stepwise linear regression results for SIS-16.

Independent variables	Beta coefficient (standard error)	95% confidence interval	t	*P*
Age	−0.226 (0.070)	−0.367 to −0.085	3.211	.002
FIM	0.658 (0.047)	0.564 to 0.751	14.080	<.001

F= 411.660 *P*<.001 Adj R2 = 0.925				

FIM: Functional Independence Measure

## DISCUSSION

In this study, we assessed HRQOL, including physical function, using the SIS-16, which is accepted as a feasible and adequate measure for assessing post-stroke function. We also investigated possible associations between demographic characteristics, clinical characteristics, cognitive function, functional status and HRQOL and identified factors that predict HRQOL. The patients in our study had mostly non-dominant hand involvement, with infarct-type stroke and left hemiparesis. Clinically, cognitive impairment and functional disability were associated with low HRQOL outcomes. In the linear regression, age and functional status showed a powerful influence on HRQOL in stroke survivors.

Several studies have shown that quality of life (QOL) in patients with stroke is worse than QOL in the general population in the first years after stroke, especially for physical factors.^5^,^19^,^20^ Cross-sectional data suggest that HRQOL and well-being after stroke are significantly impaired. A comparison of community-dwelling seniors with no prior stroke and community-dwelling stroke survivors identified a lower sense of well-being, a greater likelihood of restriction in physical and cognitive functions, worse mental health, and a greater number of comorbid health conditions in the stroke survivors compared with those without stroke.^18^ A study of 46 stroke survivors 4 years after their first stroke showed that despite a good outcome in terms of discharge from the hospital, ADL, and return to work, the HRQOL of 83% of the patients had not been restored to the pre-stroke level.^21^

In one study, the mean QOL scores decreased in the domain of physical function between 4 to 16 months after stroke and important determinants of QOL after 16 months were functional status, age and gender.^22^ Another study showed that neither age, gender, comorbidity, nor baseline disability was an important determinant of change in HRQOL from 1 to 6 months following acute stroke.^23^ Haacke et al reported a decreased HRQOL in patients 4 years after stroke and found that important determinants were physical state and cognitive impairment.^24^ Poor physical health 1 year after stroke was independently associated with being female and having diabetes mellitus, right hemispheric lesions and cognitive impairment. In another study, poor mental health 1 year after stroke was independently associated with being under 65 years, the presence of ischemic heart disease and cognitive impairment.^25^ Two studies examined the relationship between FIM and HRQOL ratings. One found high correlations between the two measures before the stroke and 12 months post-discharge.^26^ The second study found that HRQOL and FIM ratings both improved between 1 and 6 months, with modest correlations between FIM and HRQOL ratings at 6 months but not at 1 month.^27^ Sturm et al assessed HRQOL 2 years post-stroke using the Assessment of Quality of Life instrument and found disability, dementia and age as independent determinants of HRQOL in survivors.^28^ In our study, the SIS-16 values did not differ between males and females and were correlated with functional status and cognitive function. Age and the FIM score were the factors that independently predicted HRQOL.

In a previous study, multiple factors (education level, stroke type, concordance, and comorbidities) were associated with HRQOL across SIS domains. Poorer HRQOL in the physical domain was associated with more comorbidities.^29^ The presence of diabetes mellitus has previously been associated with poorer HRQOL scores.^8^ In our study, duration of education was not correlated with SIS values. There was a lack of association with sex, comorbidity, stroke type, affected side, and concordance based on SIS-16 score values. This lack of association may be attributable to the homogeneity of this sample. An alternative explanation may have been the rigorous inclusion criteria; for instance, comorbidities were all controlled and patients with comorbidities affecting their life expectancy were not included in this study.

One limitation of our study was the sample size, which was relatively small. Another issue was the inpatient rehabilitation follow-up programs, which have a strong, positive impact on HRQOL. Thus, if the study was a follow-up study instead of a cross-sectional study, more detailed results would have been gained. The last weakness may be the lack of assessment of depression and family functioning, which can be the most important determinants of HRQOL for patients after stroke.

The strength of our study was the use of a stroke-specific measure, the SIS-16, which is a comprehensive measure of health impact after stroke and adds important information regarding HRQOL. Early identification and treatment of functional disability, particularly in older stroke patients, can potentially maximize recovery and improve physical functioning. Additional data that assess depression and family functioning as additional factors may facilitate a better understanding of HRQOL. Long-term follow-up studies should preferably include comprehensive rehabilitation programs throughout the stages of stroke recovery to evaluate HRQOL in more detail.
